# A programmable, selection-free CRISPR interference system in *Staphylococcus aureus* for long-term host interaction studies

**DOI:** 10.1016/j.isci.2025.113420

**Published:** 2025-08-21

**Authors:** Roni Miah, Mona Johannessen, Morten Kjos, Christian S. Lentz

**Affiliations:** 1 Department of Medical Biology and Centre for New Antibacterial Strategies (CANS), UiT- The Arctic University of Norway, 9019 Tromsø, Norway; 2 Faculty of Chemistry, Biotechnology and Food Science, Norwegian University of Life Sciences, Ås, Norway

**Keywords:** Biological sciences, Microbiology, Biotechnology, Synthetic biology

## Abstract

Common dCas9-based CRISPR interference (CRISPRi) systems for manipulating bacterial gene expression require antibiotic selection and exogenous inducer molecules, limiting their applicability in infection models. For *Staphylococcus aureus*, we have developed a programmable, selection-free CRISPRi system leveraging the pCM29 plasmid, which is stable without antibiotic selection. In this system, dCas9 expression is regulated by an endogenous promoter, and sgRNA expression is driven by a constitutive promoter, eliminating the need for exogenous inducer molecules. We programmed the system to silence the expression of the coagulase or autolysin genes whenever their respective endogenous promoters were activated. We confirmed selection-free interference with target gene expression for ≥27 generations by qPCR and protein target-dependent *in vitro* or *in vivo* phenotypic assays (plasma coagulation, THP-1 cell, and *Galleria mellonella* infection). The system is suitable for interrogating gene function in long-term studies of *S. aureus* pathogenesis and represents a blueprint for similar CRISPRi systems in other species.

## Highlights

• ppsf-CRISPRi is functional without antibiotic selection in *S. aureus*
• ppsf-CRISPRi is driven by *S. aureus* promoter, eliminating the need for external inducers
• ppsf-CRISPRi constructs phenocopy KO-strains in long-term *in vivo* infection studies
• ppsf-CRISPRi may enable specific gene regulation in subpopulations of *S. aureus* cells

## Graphical abstract

**Figure:**
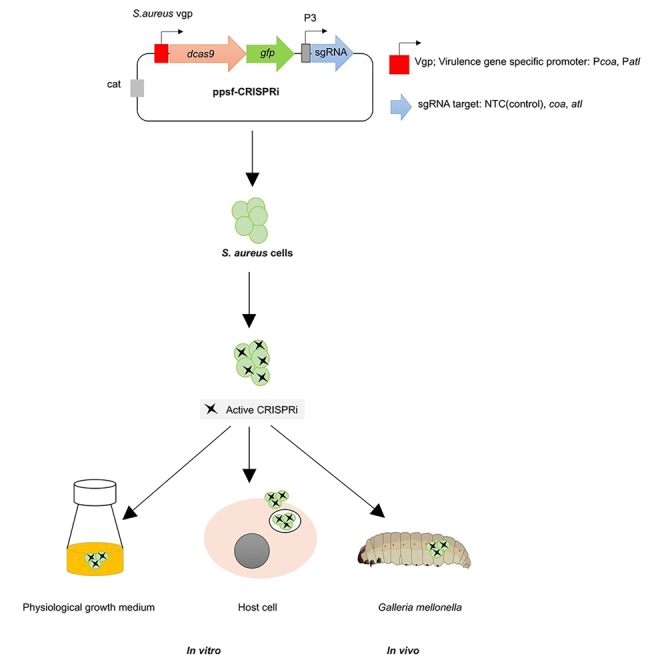

## Introduction

The unfolding antimicrobial resistance crisis urges the development of new antimicrobial treatment options for antibiotic-resistant priority pathogens, including methicillin-resistant *Staphylococcus aureus* (MRSA).^1^ *S. aureus* colonizes the nose in approx. 30% of the human population and can cause a broad range of both local and systemic, life-threatening systemic infections.^2^^,^^3^^,^^4^^,^^5^ Assigning gene function at the dynamic host-pathogen interface is critical for understanding the mechanisms of bacterial pathogenesis and evaluating potential drug targets. Traditional approaches in functional genomics of bacteria use insertion or deletion mutagenesis methods that are labour-intensive and can only be applied to non-essential genes. Recent studies have utilized CRISPR (Clustered Regularly Interspaced Short Palindromic Repeats) technologies, and particularly CRISPR interference (CRISPRi) have found wide applications in various bacterial species.^6^^,^^7^^,^^8^^,^^9^^,^^10^^,^^11^^,^^12^^,^^13^^,^^14^^,^^15^ CRISPRi gene-silencing utilizes an inactive Cas9 protein (dCas9) and customized single-guide RNA (sgRNA) complex to bind to a specific gene locus for effectively blocking transcription.^6^ The sgRNA spacer sequence of the CRISPRi tool can be easily modified to target the specific genomic regions of interest^16^^,^^17^ expanding this system’s utility in basic and applied microbial research. Furthermore, CRISPRi-seq uses pooled CRISPRi libraries and sequencing for the genome-wide quantification of gene fitness that holds significant promise for studying bacterial pathogenesis.^11^^,^^12^^,^^13^^,^^14^^,^^15^

The delivery of CRISPRi systems to bacteria has traditionally relied on replicative plasmids, requiring both antibiotic selection and exogenous inducers to achieve effective CRISPRi-based gene silencing.^6^^,^^7^^,^^8^^,^^12^^,^^13^^,^^14^^,^^15^^,^^18^^,^^19^ However, antibiotics used for selection can cause cellular changes even at subinhibitory levels, and resistance mechanisms, such as antibiotic efflux or protein mutations, come with functional costs that can affect bacterial fitness.^20^^,^^21^^,^^22^^,^^23^^,^^24^^,^^25^^,^^26^^,^^27^^,^^28^^,^^29^^,^^30^^,^^31^ Furthermore, long-term *in vivo* experiments face challenges, such as the degradation of antibiotics over time, difficulties in antibiotic delivery.^32^ Antibiotic administration to maintain plasmid stability in animal studies is unreliable, as selective antibiotic levels may not reach all infection sites and could affect the animal’s microbiota, confounding experimental results.^32^ Additionally, some antibiotics do not effectively enter host cells, hindering plasmid maintenance in intracellular bacteria.^33^^,^^34^^,^^35^^,^^36^ The same pharmacokinetic limitations associated with antibiotics and CRISPR plasmid maintenance in experimental model systems of bacterial pathogenesis and infection also apply to exogenous inducer molecules, such as IPTG (isopropyl-β-D-thiogalactopyranoside)^13^^,^^37^^,^^38^ or tetracyclines,^38^^,^^39^ which are commonly used in the classical inducible CRISPRi.^6^^,^^13^^,^^15^^,^^18^^,^^19^^,^^40^^,^^41^

One approach to eliminate the need for antibiotic selection and inducer molecules is to integrate the complete, customized CRISPRi system into the bacterial chromosome - a method known as mobile-CRISPRi.^42^ With this system, the integration of mobile-CRISPRi elements into various Gram-negative bacteria occurs via Tn7 transposons and utilizes *E. coli* as a donor strain, while Firmicutes (such as *S. aureus*), *B. subtilis* serves as a donor, and genome integration occurs via integrative and conjugative elements (ICE).^42^ Interestingly, in the Gram-negative pathogen *P. aeruginosa*, the use of weak, constitutive promoters to drive dCas9 expression has enabled the partial knock-down of essential and conditionally essential genes in longer-term infection models without an inducer.^43^^,^^44^ A single-plasmid CRISPR interference (CRISPRi) system regulated by constitutive gene promoters and operating without the need for inducers has also been reported in *S. aureus*.^45^^,^^46^ CRISPRi system has also been optimized by integrating the inducible *dcas9* cassette into the genome of *S. aureus*^47^^,^^48^ and *S. pneumoniae*.^40^ However, chromosomal integration may induce other undesired phenotypes such as impaired growth^47^ or other defects.^16^^,^^17^^,^^49^ Furthermore, the construction of chromosomal integration strains is labor intensive in many species, including *S. aureus*, limiting the upscaling of cloning process (e.g., for the construction of libraries for CRISPRi-seq). Thus, even though a variety of different CRISPRi systems have been developed over recent years (Table 1), there is still a need for complementary technologies that combine the ease-of-generation of plasmid-based, with the selection-free advantages of genome-integrated CRISPRi systems for long-term infection studies.

**Table 1:** Developed CRISPRi system in *S. aureus*

Name	Plasmid-based or genome-integrated	Promoters driving dCas9 expression	Exogenous inducer needed	Antibiotic selection	Compatibility with essential genes	Compatibility with long-term infection studies	Bacterial Species	References
Classical	One plasmid-based	P*tetO* (aTc-inducible)	Yes	Yes	Yes	Not reported	*S. aureus*	Zhao et al.^41^
Classical	Two plasmid-based	P*lac* (IPTG-inducible)	Yes	Yes	Yes	Not reported	*S. aureus*	Stamsås et al.^18^
Constitutive	One plasmid-based	P*cap* promoter (constitutive)	No	Yes	Not reported	Not reported	*S. aureus*	Dong et al.^45^
Constitutive	One plasmid-based	*rpsL* promoter (constitutive)	No	Yes	Not reported	Not reported	*S. aureus*	Chen et al.^46^
Lisbon	Two plasmid-based and genome-integrated	P*cad* (cadmium-inducible) and P*xyl*/*tetO* (tetracycline-inducible)	Yes	Yes	Yes	Not reported	*S. aureus*	Reed et al.^47^
Mobile	Genome-integrated	P*xyl*/*tetO* (tetracycline-inducible)	Yes	No	Yes	Not reported	*S. aureus* and others	Peters et al.^42^
vgp	Two plasmid-based	P*atl*, P*fnbA*, and P*coa*	No	Yes	Yes	No	*S. aureus*	Miah et al.^50^
ppsf	One plasmid-based	P*atl* and P*coa*	No	No	No	Yes	*S. aureus*	This study

Previously, we developed an inducer-free, endogenous virulence gene promoter-controlled, two plasmid-based CRISPRi system in methicillin-resistant *S. aureus*.^50^ However, as this system requires antibiotic selection, it is for that reason less suitable for *in vivo* studies. Here, we developed a programmable, plasmid-based, but selection-free (ppsf) CRISPRi system in plasmid pCM29 that is both selection-free and exogenous inducer-independent. We used *S. aureus* as a model organism for demonstrating the proof-of-concept validation of the ppsf-CRISPRi system. In this study, the system leverages the endogenous activities of the promoters of the virulence-associated genes *coa* (encoding coagulase) and *atl* (encoding autolysin) to regulate dCas9 expression.^51^^,^^52^ The retention of pCM29 plasmid without antibiotic selection enables gene silencing in the absence of external inducers and antibiotics. The system also includes a fluorescent reporter gene downstream of *dcas9* that indicates when dCas9 is expressed to activate the CRISPRi system. Proof-of-concept validation was achieved by silencing genes involved in various phenotypes and by demonstrating the system’s utility in the study of both *in vitro* and *in vivo* models of *S. aureus* infection.

## Results

### Construction of the ppsf-CRISPR interference system in *S. aureus*

Antibiotic selection is essential to maintain the activity of our IPTG-inducible two plasmid-based CRISPRi system in *S. aureus*, as demonstrated by the reduced ability to induce growth inhibition phenotype through the silencing of *pbp1* in the absence of selection pressure (Figure S1). Inspired by previous reports on the stability of pCM29 without chloramphenicol selection in *S. epidermidis* and *S. aureus* SH1000^53^ we wanted to construct a ppsf-CRISPRi system in *S. aureus* using pCM29. Attempts to clone ppsf-CRISPRi (*pbp1*) in *E. coli* IM08B at 37°C resulted in a truncation of the *dcas9* gene from 4105 to 559 bp, as confirmed by whole plasmid sequencing. This modification, likely due to dCas9-related toxicity as previously reported,^50^ rendered the constructs non-functional in *S. aureus* (Figure S2). However, the construct could be successfully created when the cloning was performed at 27°C. At this lower temperature, the expression of the *S. aureus* gene promoters in *E. coli* was drastically reduced, and there was no evidence of dCas9-based toxicity (Figure S3). Thus, we were able to construct stable ppsf-CRISPRi plasmids in *E. coli* IM08B (Figure S4; Table S1) that were directly transformed into *S. aureus* USA300 LAC strains for functional analysis.

### Evaluation of pCM29-plasmid backbone retention in *S. aureus* without antibiotic selection

**Figure 1:**
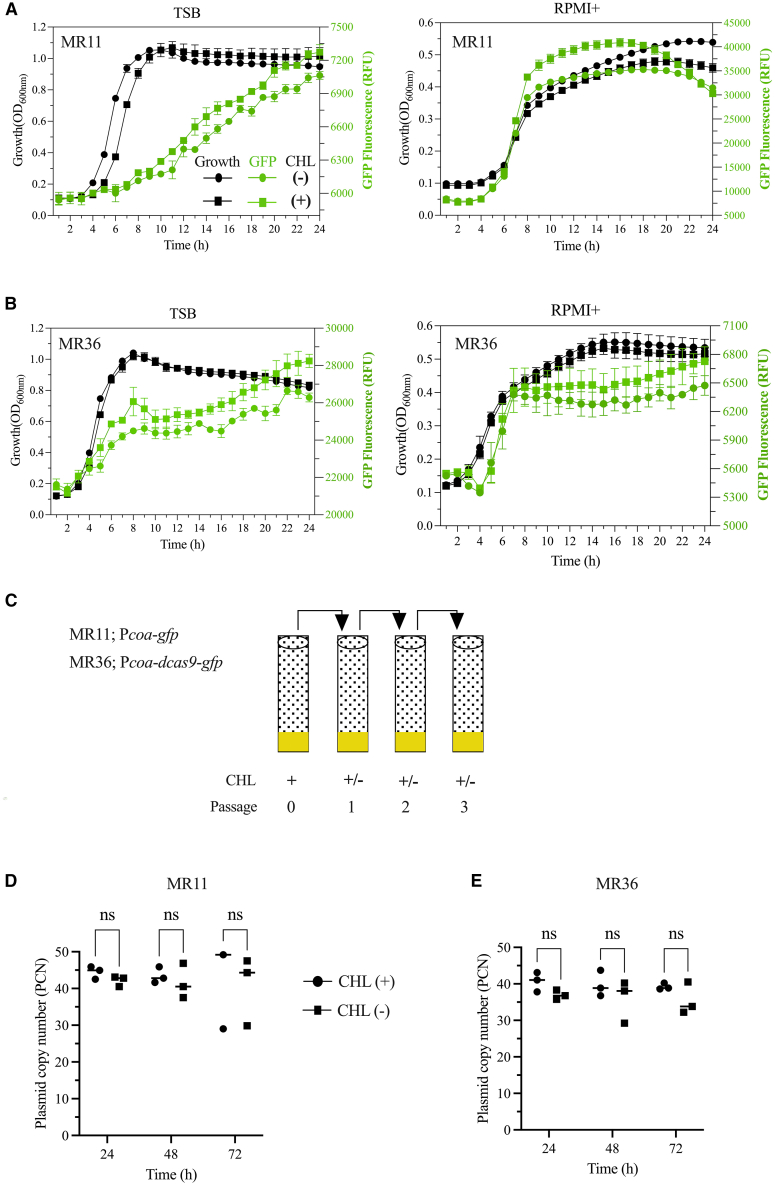
Retention of pCM29 plasmid in *S. aureus* USA 300 LAC without antibiotic selection (A and B) Overlays of growth curves (OD600) and GFP fluorescence levels (in RFU, i.e., relative to the internal standard in the instrument) of *S. aureus* fluorescence reporter strains, MR11 (A, carrying pCM29-P*coa*-*gfp*) and ppsf-CRISPRi strain, MR36 (B, carrying pCM29-P*coa*-*dcas9*-*gfp*). Cultures were grown either in TSB or cell culture medium RPMI+ with (+) and without (−) chloramphenicol (CHL). (C) Schematic representation of serial culture passage with 24-h intervals for the MR11 and MR36 strains. Cultures were initially grown in TSB medium with CHL and then passaged serially in TSB medium with (+) and without (−) CHL. (D and E) The copy numbers of pCM29-P*coa*-*gfp* in MR11 (D) and pCM29-P*coa*-CRISPRi(*coa*) in MR36 (E) per cell were determined using qPCR across three serial passages of cells cultured in the presence or absence of CHL. Data show means ± SD of *n* = 3 biological replicates (each recorded with three technical replicates). Statistical significance was assessed using two-way ANOVA. A *p*-value <0.05 was considered statistically significant; ns is non-significant.

To evaluate whether the ppsf-CRISPRi constructs are stable without antibiotic selection, we first used the plasmid-encoded fluorescence reporter as a proxy for plasmid stability and monitored fluorescence over 24h of growth in TSB and RPMI+, a common mammalian cell-culture medium, in the presence and absence of chloramphenicol. GFP fluorescence in ppsf-CRISPRi strain MR36, as well as in fluorescence reporter strain MR11 (Table 2) remained stable in the absence of chloramphenicol in both media (Figures 1A and 1B). To confirm the long-term presence of the plasmid, we determined the plasmid copy number across three serial culture passages with 24-h intervals (equivalent to 27 generation times), both with and without chloramphenicol in TSB by qPCR (Figure 1C). The results indicate that both MR11 and MR36 strains maintained a consistent number of plasmids per cell, regardless of the presence or absence of chloramphenicol over the passage of culture days (Figures 1D and 1E). In conclusion, these results suggest the long-term stability of the pCM29-plasmid backbone in *S. aureus* without antibiotic selection for at least 27 generations.

**Table 2:** Bacterial strains used in this study

Bacterial strains	Genotype or description	Source or references
**E. coli**		

IM08B	DH10B derivative, *Δdcm, Phelp-hsdMS, PN25-hsdS* (strain expressing the *S. aureus CC8* specific methylation genes)	Monk et al.^54^
MR4	IM08B carrying pCM29-P*coa*-*gfp*, ampᴿ	Miah et al.^50^
MR33	IM08B carrying ppsf-CRISPRi∗(*pbp1*), ampᴿ	This study
MR34	IM08B carrying ppsf-CRISPRi (*pbp1*), ampᴿ	This study

**S. aureus**		

USA300_JE2	A plasmid-cured derivative of USA300_LAC and parent strain of Nebraska Transposon Mutant Library	Fey et al.^55^
JE2 (*atl*::Tn)	JE2 *atl* transposon mutant from Nebraska library	Fey et al.^55^
USA300_LAC	Wild-type, coagulase positive, community-acquired MRSA clone from the USA300 lineage, isolated from Los Angeles County (LAC)	Voyich et al.^56^
MR11	LAC carrying pCM29-P*coa*-*gfp*, cmᴿ	Miah et al.^50^
MR15	LAC carrying pLOW-P*lac*-*dcas9* and pVL2336-sgRNA(*pbp1*), ermᴿ, cmᴿ	Miah et al.^50^
MR16	LAC carrying pLOW-P*lac*-*dcas*9 and pCM29-sgRNA(*pbp1*), ermᴿ, cmᴿ	Miah et al.^50^
ppsf-CRISPRi strains	LAC carrying pCM29 with *dcas9*, *gfp* downstream of coagulase or autolysin gene promote and sgRNA expression cassette under constitutive P3 promoter	–
MR35	P*coa*-CRISPRi (NTC), cmᴿ	This study
MR36	P*coa*-CRISPRi (*coa*), cmᴿ	This study
MR37	P*coa*-CRISPRi (*atl*), cmᴿ	This study
MR38	P*coa*- TruncCRISPRi (*pbp1*), cmᴿ	This study
MR39	P*atl*-CRISPRi (NTC), cmᴿ	This study
MR40	P*atl*-CRISPRi (*atl*), cmᴿ	This study

ampᴿ, ampicillin resistance; cmᴿ, chloramphenicol resistance; ermᴿ, erythromycin resistance; NTC, non-target control (sgRNA cassette containing a 20-nucleotide base-pairing region derived from a non-targeting control sequence originally designed to target the luciferase (*luc*) gene)^18^; Trunc, truncated *dcas9*.

### Ppsf-CRISPR interference system allows specific and robust interference with *coa* gene expression and coagulase function

We next aimed to evaluate whether the ppsf-CRISPRi system could effectively target specific gene expression and related cellular phenotypes even in the absence of antibiotic selection. As a model target, we chose coagulase, which is a critical virulence factor responsible for converting fibrinogen to fibrin, thereby causing plasma clotting.^52^^,^^57^ To assess the repression of coagulase transcription and function, we used the ppsf-CRISPRi strain MR36 with *coa* as the sgRNA target and MR35 as a non-targeting control (NTC) (Table 2). In both strains, dCas9 expression was regulated by the coa promoter. Both MR35 and MR36 were subjected to three consecutive culture passages at 24-h intervals in TSB and RPMI+ media, with and without chloramphenicol (Figure 1C), prior to assessing the expression of *coa* using qPCR.

**Figure 2:**
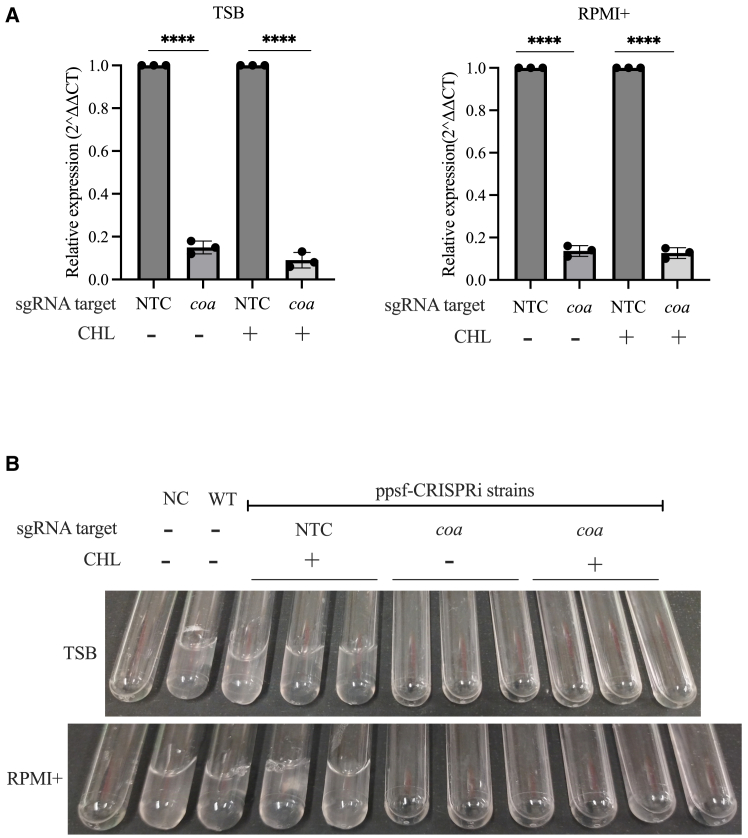
Silencing of *coa* expression and plasma coagulation using the ppsf-CRISPRi system (A) The repression of *coa* mRNA of the indicated ppsf-CRISPRi strains, third passage grown in TSB or RPMI+ with (+) and without (−) chloramphenicol (CHL). The sgRNA target used was *coa*. A non-targeting sgRNA was included as a control (NTC). dCas9 expression was regulated by the coa promoter. qPCR was performed on exponential cell cultures (OD_600_ of 1) harvested after passage three. The relative coagulase expression was calculated after normalization by 16S rRNA (*rrsA*). The data represent the mean ± SD of *n* = 3 biological replicates, each recorded with two technical replicates. Significance was tested for each construct against its NTC control pair by unpaired, two-tailed Student’s t-test. Significant differences were defined as ∗∗∗∗*p* < 0.0001. (B) The figure shows photographs of coagulase test tubes 4 h after adding indicated third passage ppsf-CRISPRi strains to rabbit plasma. The coherent clot formation is indicative of coagulation (arrowheads). Three tubes for each strain are representative of three biological replicates. The negative control (NC) contained medium (TSB or RPMI+) with rabbit plasma, and WT represents the *S. aureus* USA 300 LAC strain with rabbit plasma.

The results showed that the *coa*-targeting MR36 strain exhibited a significant reduction in *coa* expression compared to the control strain MR35, regardless of the growth medium and presence or absence of chloramphenicol (Figure 2A). The sustained silencing of the *coa* gene in strain MR36 (even after three 24-h passages in the absence of selection) led to a functional deficiency in the coagulation of rabbit plasma (Figure 2B), suggesting that coagulase activity was efficiently abrogated. In contrast, clotting occurred in both the CRISPRi control strain (MR35) and the WT strains. This demonstrates that the ppsf-CRISPRi system can robustly silence virulence genes such as *coa*, effectively interfering with their biological function in *S. aureus* without the need for antibiotic selection.

### Ppsf-CRISPRi system’s functionality in studying the *S. aureus* infection of human cell line

**Figure 3:**
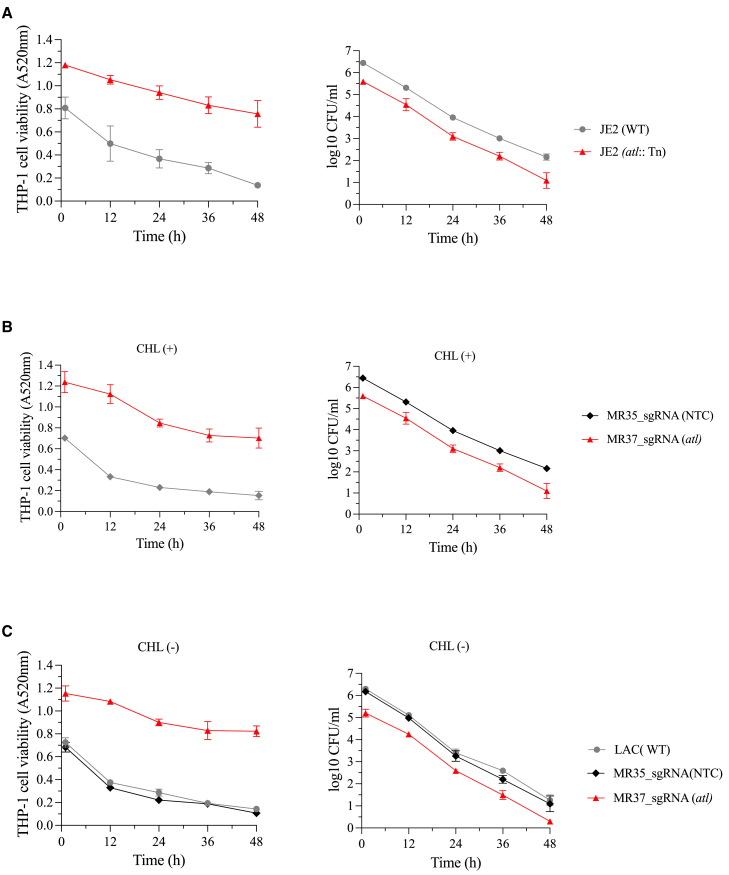
ppsf-CRISPRi system to study THP-1 macrophage infection with *S. aureus* (A–C) Time-course THP-1 cell viability (MTT) (left panel) and infection assay (right panel) following infection with the indicated *S. aureus_*JE2 or LAC WT, transposon mutant JE2 (*atl*:Tn), and ppsf-CRISPRi control strain (MR35, NTC) and strains targeting *atl* (MR37). For ppsf-CRISPRi constructs, the sgRNA target was *atl* or an NTC derived from the luciferase gene, dCas9 expression controlled by the coa promoter. Before the infection assay, the indicated ppsf-CRISPRi strains were grown in TSB medium with CHL. They were then serially passaged in TSB medium with (+) and without (−) CHL at 24-h intervals for up to three culture passages. Indicated WT and transposon mutant strains were also passaged similarly to the ppsf-CRISPRi strains without CHL. Following the final culture passage, the (+) CHL and (−) CHL cultivated strains were subjected to the time-course THP-1 infection in the presence and absence of CHL, respectively. The data represent the mean ± SD of *n* = 3 biological replicates.

To evaluate the utility of the ppsf-CRISPRi system for studying *S. aureus* infection in human cell lines, we monitored the intracellular survival of bacteria in the human THP1 macrophage cell line, as well as THP1 cell viability over 48 h. As a model target, we chose to interfere with the *atl* gene, which encodes autolysin (Atl), a peptidoglycan hydrolase critical for cell wall degradation and division.^58^ The strains used included WT *S. aureus* USA300 LAC and two ppsf-CRISPRi constructs: one targeting *atl* (MR37) and a non-targeting control (NTC, MR35), both with dCas9 expression driven by the coa promoter (Table 2). Additionally, WT *S. aureus* USA300_JE2 and its *atl* transposon mutant strain were used as controls. The ppsf-CRISPRi strains were subjected to the same 3-passage pre-cultivation in the presence/absence of antibiotic selection described earlier (Figure 1C), and THP-1 cell infection experiments were carried out in the presence or absence of chloramphenicol (10 μg/mL). This concentration of chloramphenicol, which is routinely used for plasmid maintenance, did not affect THP-1 cell viability (Figure S5), consistent with previous reports in H1299 cells.^59^ The viability of macrophages infected with WT or non-targeting ppsf-CRISPRi control strain MR35 decreased markedly over the time, whereas macrophages infected with *atl*-deficient strains (both transposon mutant and ppsf-CRISPRi knockdown) had significantly higher viability (Figures 3A–3C, left panel). We observed that the levels of viable intracellular bacteria (CFUs) were reduced approx. 10-fold in the *atl* transposon mutant compared to the WT throughout the assay period (Figure 3A, right panel). Similarly, regardless of the presence or absence of chloramphenicol in the pre-cultivation period and throughout the experiment, the *atl*-targeting ppsf-CRISPRi strain MR37 exhibited a similar 1-log reduction of intracellular CFU when compared to WT and NTC strains (Figures 3B and 3C, right panel).

Since the difference in intracellular CFU between *atl*-deficient and control strains manifests already at 1 h and the kinetics of the decline in CFU over time is similar across all strains, our data suggest that these outcomes are not due to reduced intracellular viability or faster killing of *atl*-deficient bacteria, but rather reflect reduced internalization. This finding aligns with previous research demonstrating that *S. aureus* autolysin mutant strains exhibit decreased binding to host cell ligands and reduced internalization into host cells.^51^^,^^60^ Collectively, these results support the effectiveness of the ppsf-CRISPRi system in studying *S. aureus* infections in host cell infection or co-culture experiments without selection.

### The ppsf-CRISPRi system is functional during *Galleria mellonella* infection

**Figure 4:**
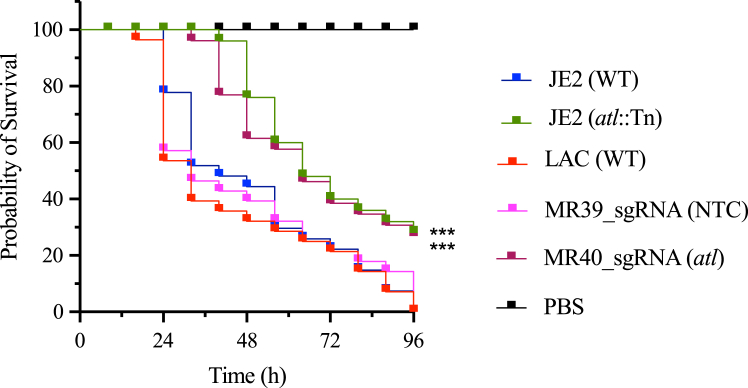
ppsf-CRISPRi system for studying *Galleria mellonella* infection with *S. aureus* Kaplan–Meier (KM) survival plots of *G. mellonella* larvae after inoculation with indicated *S. aureus* WT, transposon mutant, and ppsf-CRISPRi strains. For ppsf-CRISPRi strains, dCas9 expression was controlled under the autolysin gene promoter (P*atl*). Before the *G. mellonella* infection study, the ppsf-CRISPRi strains were cultured in TSB medium with CHL and then passaged up to three times in antibiotic-free TSB medium at 24-h intervals. Indicated WT and transposon mutant strains were also passaged similarly to the ppsf-CRISPRi strains. The plots indicate the average of *n* = 3 independent biological replicates (i.e., bacterial cultures originating from 3 different colonies), that were each used to infect 10 larvae per group. Mortality was monitored every 8 h for 96 h (*N* = 180). Larvae injected with PBS served as a negative control. The asterisks in the graph indicate a significant difference in the survival of autolysin (*atl*) transposon mutant strain compared to their wild-type (WT) strain and *atl* silenced CRISPRi strain compared to their non-target CRISPRi control (NTC) or wild-type (WT) strain. Pairwise comparisons of survival curves were performed using the log rank (Mantel-Cox) test. Significant differences were defined as ∗∗∗*p* < 0.001.

To evaluate the utility of the ppsf-CRISPRi system for studying *S. aureus* virulence in an *in vivo* setting, we used the *G. mellonella* infection model. *G. mellonella* larvae were infected with a atl promoter-driven ppsf-CRISPRi strain targeting *atl* (MR40) and a non-targeting control strain (MR39) (Table 2). Wild-type USA300_JE2 or LAC strains and corresponding *atl* transposon mutant were included as controls. Larval survival was monitored over 96 h. Infections with either the WT strain or the non-targeting ppsf-CRISPRi control (MR39) resulted in 100% mortality within 96 h (Figure 4). In contrast, larvae infected with the *atl* transposon mutant or the ppsf-CRISPRi *atl* knockdown strain (MR40) had a significantly higher survival rate. Approximately 30% of the larvae infected with these strains were still alive after 96 h (Figure 4). This result shows that disrupting the *atl* gene function, either by transposon mutation or by CRISPRi knockdown, reduces the virulence of *S. aureus*, leading to increased larval survival. These data also demonstrate that the ppsf-CRISPRi system is indeed efficient in studying *S. aureus* infection in the *G. mellonella* infection model without relying on antibiotic selection.

## Discussion

CRISPRi-based approaches hold promise for the identification of virulence factors critical for bacterial pathogenesis and understanding the bottlenecks that pathogens encounter during infection.^13^ However, the widely used plasmid-based inducible CRISPRi system requires antibiotic selection for plasmid maintenance and inducer molecules for dCas9 expression. While functional, this classical system carries drawbacks for *in vivo* studies of bacterial pathogenesis due to its reliance on inducers and antibiotics, which can impact bacterial fitness and experimental outcomes. We explored an alternative approach for inducer independent and selection free CRISPRi system development. Previously, we developed an endogenous virulence gene promoter (vgp)-controlled CRISPR interference (vgp-CRISPRi) system in *S. aureus*.^50^ This system did not require external inducers, but still relied on antibiotic selection, similar to the classical inducible CRISPRi system. Despite the progress made with this vgp-CRISPRi system, the need for antibiotics introduced potential challenges for applying this system for *in vivo* studies of *S. aureus*. Inspired by previous reports on the stability of pCM29 plasmid without chloramphenicol selection in *S. epidermidis* and *S. aureus* SH1000,^53^ we have developed the pCM29-based ppsf-CRISPRi system in the MRSA *S. aureus* strain, which was retained and active in *S. aureus* even in the absence of antibiotic selection for at least 27 generation times.

How can the ppsf-CRISPRi system be programmed? In the current study, the constructs were benchmarked against and designed to mimic knock-out strains. By placing dCas9 expression under control of the promoter that also controls the target of the sgRNA (here *coa* or *atl*), our constructs were programmed to interfere with the expression of these virulence genes, whenever their promoter is activated. The application of the ppsf-CRISPRi system was successfully validated for both *in vitro* and *in vivo* studies of *S. aureus* without selection. Of note, in all assay systems, the performance of the ppsf-CRISPRi system phenocopied the effects of the transposon mutants, demonstrating that it is a viable alternative to studies using knock-out strains.

An important practical consideration for future applications is the feasibility of strain construction. During the generation of this ppsf-CRISPRi construct, we encountered dCas9 toxicity-dependent plasmid modification issues in *E. coli* due to the expression of *dcas9* from the *S. aureus* gene promoter at 37°C. The molecular mechanism behind this dCas9 toxicity is unknown, but it may be related to the previously reported “bad-seed” sequence.^61^ Rostain et al.^61^ demonstrated that when dCas9 is expressed along with guide RNAs carrying bad-seed sequences, it could bind to hundreds of off-target positions in *E. coli* bacterial genomes, leading to silencing and toxicity. Plasmid modification caused by dCas9 toxicity in the cloning host *E. coli* remains a major challenge in designing customized CRISPRi circuits, as we have observed. Our findings underscore an important consideration for developing single-plasmid CRISPRi systems controlled by endogenous gene promoters in any target strains when *E. coli* is used as an intermediate cloning host. It is critical to verify whether the endogenous promoter driving dCas9 expression is active in *E. coli*. Previously, we reported that all four tested *S. aureus* promoter sequences (P*coa*, P*atl*, P*sar*A P1, and P*fnb*A) could drive the expression of GFP in *E. coli* from pCM29-derived plasmids to different levels.^50^ If the promoter-of-interest is indeed active, strategies to reduce its activity and minimize dCas9 expression are essential to prevent dCas9 expression at toxic levels. By lowering the cloning temperature, we succeeded in mitigating the dCas9 toxicity-dependent plasmid modification issues and generated stable ppsf-CRISPRi constructs in *S. aureus*. To facilitate the construction of desired CRISPRi strains in just a single cloning step and enhance the flexibility and efficiency of sgRNA cloning, the incorporation of Golden Gate cloning sites into the ppsf-CRISPRi construct is required.

In addition to gene silencing, the ability to monitor endogenous gene promoter activity using a fluorescent reporter downstream of the *dcas9* gene provides a dual-function CRISPRi tool for studying both gene regulation and promoter dynamics under different environmental conditions. In theory, if gene promoters that control *dcas9* are only activated under specific conditions e.g., during infection, the activation of the CRISPRi system would then also be restricted to those conditions. Similarly, cell-to-cell heterogeneity in gene expression and promoter activity could be exploited for the specific targeting of bacterial subpopulations. We anticipate that the use of promoters that are activated heterogenously across different cells within a cell population will enable a pathway for the functional manipulation of cellular subpopulations. Suitable promoters and induction conditions for both conditional activation and in cellular subpopulations remain to be determined in future studies. Furthermore, we envision that the use of either conditionally active endogenous promoters (as in this study) or weak constitutive promoters for dCas9 expression^43^^,^^44^ in combination with broad-host-range, antibiotic-selection-free plasmids^62^ will allow for facile, programmable inducer-free interrogation of gene function across diverse bacterial species without the complications of chromosomal integration.

In conclusion, the ppsf-CRISPRi system is a versatile and efficient tool for exploring gene expression and regulation in *S. aureus* without the limitations associated with chromosomal integration, inducers, and antibiotic selection. We believe that the adaptability and flexibility of the ppsf-CRISPRi approach will enable precise, programmable gene silencing within specific bacterial species in a complex microbiome, offering new insights into the functional dynamics of bacterial interactions at the host interface.

### Limitations of the study

One of the advantages of inducible CRISPRi is that it allows the inducible knock-down of essential genes.^18^^,^^40^^,^^41^^,^^47^ In its current form, the ppsf-CRISPRi system is not suitable for the interrogation of essential genes. However, this limitation could be overcome by the use of weak, constitutive promoters to drive dCas9 expression.^43^^,^^44^ Alternatively, the ppsf-CRISPRi system could be adapted for targeting essential genes and critical pathways if the promoter driving dCas9 is specifically conditionally activated upon encountering specific environmental cues during host infection that are absent during routine *in vitro* cultivation.

## Resource availability

### Lead contact

Further information and requests for resources should be directed to and will be fulfilled by the lead contact, Christian S. Lentz (christian.s.lentz@uit.no).

### Materials availability

Bacterial strains generated in this study will be made available on request.

Plasmids generated in this study have been deposited in Addgene, deposition ID https://www.addgene.org/depositing/86181/. see Table S1.

### Data and code availability

• Data: Data reported in this article will be shared by the lead contact upon request.
• Code: This article does not report original code.
• Additional information: Any additional information required to reanalyze the data reported in this article is available from the lead contact upon request.

## Acknowledgments

This work was funded by a Center for New Antibacterial Strategies (CANS) starting-grant through the Trond-Mohn Foundation to C.S.L. M. K. is supported by a grant from JPIAMR (Research Council of Norway grant 296906). We would like to thank Simen Hermansen, NMBU, for the structure analysis of mutated dCas9 variants. We would also like to express our gratitude to Bhupender Singh and Julia Maria Kloos, UiT, for their assistance in coordinating with Eurofins Genomics for the whole plasmid sequencing and Kjersti Julin, UiT for assistance with human cell culture.

## Author contributions

Experiment design: All authors, data acquisition: R.M., data analysis: All authors, original article draft: R.M., article writing: all authors. Concept: R.M. and C.S.L. Funding: C.S.L.

## Declaration of interests

The authors declare no conflict of interest.

## STAR★Methods

### Key resources table

**Table:** 

REAGENT OR RESOURCE	SOURCE	IDENTIFIER
**Bacterial and virus strains**		

*E. coli* and *S. aureus* WT, along with their genetically engineered strains	Refer to Table 2	N/A

**Biological samples**		

Rabbit Plasma	Bio-Rad	Cat NO: 56352
Fetal bovine serum (FBS)	Sigma-Aldrich	Cat NO: F7524

**Chemicals, peptides, and recombinant proteins**		

Standard Agarose - Type LE	BioNordika	Cat NO: BN-50002
1 Kb Plus DNA Ladder	Invitrogen	Cat NO: 10787018
PBS	Sigma	Cat NO: D8537
Calcium chloride	Sigma-Aldrich	Cat NO: C1016
Glycerol 86%	VWR Chemicals	Cat NO: 24385.295
Sucrose	Sigma-Aldrich	Cat NO: 16104
Triton™ X-100	Sigma-Aldrich	Cat NO: T8787
Ampicillin	Sigma-Aldrich	Cat NO: A9393
Chloramphenicol	Sigma-Aldrich	Cat NO: C0378
Erythromycin	Sigma-Aldrich	Cat NO: E5389
Gentamicin	Sigma-Aldrich	Cat NO: G1397
IPTG	Sigma-Aldrich	Cat NO: 16758
Ethanol 96% (v/v)	VWR Chemicals	Cat NO: 20823.362
RNAprotect Bacteria Reagent	QIAGEN	Cat NO: 76104
Lysostaphin	Sigma-Aldrich	Cat NO: L7386
Lysozyme	Thermo Scientific™	Cat NO: 89833
DNase I	Sigma-Aldrich	Cat NO: AMPD1
Bsu36I	New England Biolabs	Cat NO: R0524S
T4 DNA Ligase	New England Biolabs	Cat NO: M0202S
1% penicillin/streptomycin	Gibco™	Cat NO: 11548876
Phorbol 12-myristate 13-acetate	Sigma-Aldrich	Cat NO: P8139
MTT (3-(4,5-dimethylthiazol-2-yl)-2,5-diphenyltetrazolium bromide reagent)	Invitrogen	Cat NO: M6494
Dimethyl sulfoxide (DMSO)	Sigma-Aldrich	Cat NO: 472301

**Critical commercial assays**		

BigDye™ Terminator v3.1 Cycle Sequencing Kit	Applied Biosystems™	Cat NO: 4337455
Wizard® Genomic DNA Purification Kit	Promega	Cat NO: A1120
E.Z.N.A. Plasmid DNA Mini Kit I (Q – spin)	Omega	Cat NO: D6942
Phusion™ High-Fidelity DNA Polymerases	Thermo Scientific™	Cat NO: F530S
DreamTaq PCR Master Mixes (2X)	Thermo Scientific™	Cat NO: K1081
MagExtractor -PCR & Gel Clean up	Toyobo	Cat NO: NPK-601
ZymoBIOMICS DNA miniprep kit	Zymo Research	Cat NO: D4304
PowerTrack SYBR Green Master Mix	Applied Biosystems™	Cat NO: A46012
RNeasy Mini Kit	QIAGEN	Cat NO: 74104
High-Capacity cDNA Reverse Transcription Kit	Applied Biosystems™	Cat NO: 4368814

**Deposited data**		

Plasmid DNA sequence	Refer to Table S1	Addgene: deposition ID https://www.addgene.org/depositing/86181/. Click or tap if you trust this link.">86181

**Experimental models: Cell lines**		

THP-1 cell	ATCC	Cat NO: TIB-202 ™

**Experimental models: Organisms/strains**		

*Galleria melonella* larvae	Reptilutstyr AS (Norway)	N/A

**Oligonucleotides**		

Primer sequences	Refer to Table S2	N/A
sgRNA sequences	Refer to Table S3	N/A

**Recombinant DNA**		

Recombinant plasmids	Refer to Table S1	N/A

**Software and algorithms**		

Excel	Microsoft 365	N/A
Prism 10	GraphPad Software, Inc.	N/A
LightCycler® 96 system software version 1.1	Roche Diagnostics	N/A

### Experimental models and subject details

#### Cell lines

The human macrophage cell line THP-1 cells (TIB-202, ATCC) were cultured in RPMI+ media supplemented with 1% penicillin/streptomycin (Gibco™). Cells were authenticated based on cellular morphology assessed by microscopy and confirmed to be free of mycoplasma contamination. Before the infection assay, THP-1 monocyte differentiation was initiated by incubating the cells with 10 ng/mL phorbol 12-myristate 13-acetate (Sigma-Aldrich) for 2 days at 37°C in a 5% CO_2_ environment. Following this 2-day differentiation period, the culture medium was aspirated, and the cells were washed three times with PBS. Subsequently, the cells were further incubated for 1 day in a fresh RPMI+ medium (supplemented with 10 μg/mL CHL when appropriate).

#### Bacterial strains

The *S. aureus* and *E. coli* strains used in this study are listed in Table 2. The routinely used growth media were Lysogeny Broth (LB) or LB agar for *E. coli*, and Tryptic Soy Broth (TSB) or Tryptic Soy Agar (TSA) for *S. aureus*, with incubation at 37°C and shaking at 220 rpm. *S. aureus* cells initially grown in TSB were subsequently cultured in RPMI+ medium (RPMI-1640 supplemented with 10% FBS), if appropriate, since we had previously observed that the coa promoter activity was significantly higher in RPMI+ compared to TSB,^50^ we here included RPMI+ as a growth medium to trigger robust expression of dCas9.

#### Animals

*Galleria mellonella* larvae were obtained from Reptilutstyr AS (Norway). Upon arrival, the larvae were stored at 12 °C and used within 7 days. Ten randomly selected larvae were used per group. Sex and age of the larvae were not determined.

### Method details

#### Plasmid transformation and maintenance procedures

Chemically competent *E. coli* IM08B cells were prepared according to Chang Y et al., 2017^63^ and routinely used for transformation of the constructed plasmid according to standard heat shock protocol.^64^ *S. aureus* USA 300 LAC was transformed with plasmid DNA isolated from IM08B^54^ by electroporation. Preparation of *S. aureus* electrocompetent cells and electroporation were performed as described before.^65^ Ampicillin was used at a final concentration of 100 μg/ml in LB broth or agar plates for the selection of recombinant plasmid-transformed *E. coli* colonies. Chloramphenicol (10 μg/ml) was used for the selection of recombinant pCM29-backbone plasmid-transformed *S. aureus* strains*.* Erythromycin (5 μg/ml) and chloramphenicol (10 μg/ml) were used for maintenance of pLOW and pVL2336 backbone plasmids, respectively, in *S. aureus* strains. IPTG was used at 250 μM (final concentration) for *lac*-promoter-induced dCas9 expression in the classical CRISPRi system.

#### Generation of ppsf-CRISPRi constructs

To generate ppsf-CRISPRi constructs, the *dcas9* gene was amplified from pLOW-P*lac*-*dcas9*^18^ using the primer sets RM 27/28 (Table S2). The amplified fragment was digested with Bsu36I (New England Biolabs) and ligated into the corresponding site of a previously constructed pCM29-based sgRNA expression vector^50^ using T4 Ligase (New England Biolabs). The ligation mixture was then transformed into *E. coli* IM08B cells, plated on LB agar containing 100μg/ml ampicillin, and incubated overnight at either 27°C or 37°C. The correct construct was confirmed through restriction mapping, PCR, and whole plasmid sequencing. This resulted in the ppsf-CRISPRi constructs with *pbp1*, *coa*, *atl,* and non-targeting control (NTC) as the sgRNA (Table S1). In these constructs, the expression of *dcas9* and *gfp* is regulated by the virulence gene promoters of coagulase (*coa*) and autolysin (*atl*), while sgRNA expression is driven by a constitutive P3 promoter. The sequences for the sgRNA base-pairing regions are listed in Table S4.

#### Bacterial growth and GFP fluorescence analysis

Bacterial growth (OD_600_) and GFP fluorescence were measured in a microplate assay on a Synergy H1 Hybrid Reader (BioTek). *S. aureus* strains were grown overnight with shaking at 37°C, and *E. coli* strains were grown at 37 or 27°C as specified. Cell cultures were diluted to an OD_600_ of 0.1 in their respective fresh medium (TSB or RPMI+ for *S. aureus* and LB for *E. coli* strains, respectively). Two μL of these diluted overnight cultures were inoculated into 298 μL of each respective fresh medium in a 96-well flat-bottom polystyrene tissue culture plate (Falcon). The plate was incubated directly in a Synergy H1 Hybrid Reader (BioTek) microtiter plate reader at 37 or 27°C as specified with constant double orbital shaking (400 rpm for 5 s) in-between measurements. Optical density (OD_600_) and GFP fluorescence (excitation 479, emission 520) were measured at 1-hour intervals for up to 24 hours. GFP fluorescence was recorded as relative fluorescence units (RFU, i.e. relative to the internal standard in the instrument). The OD_600_ and GFP fluorescence values from 3 biological replicates that were each derived from 3 technical replicates were averaged and corrected from blank wells containing only medium for each strain.

#### Plasmid copy number determination by quantitative PCR (qPCR)

*S. aureus* ppsf-CRISPRi strain, MR36 and the previously constructed fluorescence reporter strain MR11 (Table 2) were initially grown in TSB medium with chloramphenicol (CHL). Cultured cells were then subjected to a three-step serial culture process in TSB with 24-hour intervals (denoted passages 1,2 and 3) in the presence or absence of CHL. From each passage, samples were harvested and subjected to total DNA isolation using the ZymoBIOMICS DNA miniprep kit (Zymo Research) following the manufacturer’s instructions and subjected to real-time PCR assay performed with PowerTrack SYBR Green Master Mix (Applied Biosystems™) containing 0.5 μM each primer set. After cycling, melt curves analysis was performed between 70°C and 90°C. All quantitative PCR (qPCR) data were analyzed using LightCycler® 96 system software version 1.1 (Roche Diagnostics). The plasmid copy number per cell was defined as the ratio of the plasmid to chromosomal DNA copies and was calculated using the formula 2^-ΔCq^, where -ΔCq represents the difference in quantification cycles between the plasmid gene (*gfp*) and the chromosomal gene (*groEL*).^66^ The qPCR primers are listed in Table S2 qPCR was performed in three technical replicates from their three biological replicates.

#### RNA purification, reverse transcription, quantitative PCR (qPCR), and coagulase test

*S. aureus* ppsf-CRISPRi (NTC) strain MR35 and the ppsf-CRISPRi (*coa*) strain MR36 (Table 2) were grown in TSB medium with CHL. Cultured cells were then subjected to a three-step serial culture process in TSB and RPMI+ medium with 24-hour intervals (denoted passages 1,2 and 3) in the presence or absence of CHL. Exponential cell cultures (OD_600_ of 1) from passage 3, were harvested and subjected to total mRNA isolation. Harvested cells were diluted in PBS (D8537, Sigma) and adjusted to McFarland 0.5 (i.e about 10^8^ cells/ml). Subsequently, cells were treated with 2× volume of RNAprotect Bacteria Reagent (QIAGEN) for 10 min at room temperature and collected as pellets by centrifugating for 10 min at 5,000×g. Lysostaphin, 1 μg/μL, and lysozyme, 10 μg/μL were added as final concentration to the pellets and subsequently prepared suspension was incubated at 37 °C for 30 minutes for lysis of bacterial cells before RNA isolation. RNA extraction was performed using a RNeasy Mini Kit (QIAGEN). The remaining DNA in the isolated RNA was degraded by DNase I (Sigma-Aldrich). DNA-free RNA was subjected to reverse transcription (RT) with the High-Capacity cDNA Reverse Transcription (RT) Kit (Applied Biosystems™). RNA extraction, DNase treatment, and RT were performed according to the manufacturer’s direction. Synthesized cDNA solution was 10,000-fold diluted with RNase-free water, and 2μl of the diluted cDNA solution was subjected to real-time PCR assay performed with PowerTrack SYBR Green Master Mix (Applied Biosystems™) containing 0.5 μM each primer set. After cycling, melt curves analysis was performed between 70°C and 90°C. All quantitative PCR (qPCR) data were analyzed using LightCycler® 96 system software version 1.1(Roche Diagnostics). Relative coagulase expression was calculated according to the 2^−ΔΔCt^ method after normalization by 16SrRNA (rrsA). The qPCR primers are listed in Table S2. qPCR was performed in three technical replicates from their three biological replicates.

For the coagulase test, exponential cell cultures (OD_600_ of 1) from passage 3 were diluted in PBS and adjusted to McFarland 0.5. Subsequently, 0.5 ml of each McFarland-adjusted culture was added to 0.5 ml of rabbit plasma (Bio-Rad). The samples were incubated in a water bath at 37°C for 4 h. The level of coagulation was verified by tipping the tubes to a 45° angle. A negative control (NC) sample contained medium only. A test was considered positive if the plasma in the tube formed a coherent clot. All experiments were repeated three times as independent biological replicates to examine the reproducibility.

#### Time-course THP-1 infection and cytotoxicity assay

For THP-1 infection and cytotoxicity assay, *S. aureus* ppsf-CRISPRi (NTC) strain MR 35 and the ppsf-CRISPRi (*atl*) strain, MR37 (Table 2) were grown in TSB medium with CHL. Cultured cells were then subjected to a three-step serial culture process in TSB medium with 24-hour intervals (denoted passages 1,2 and 3), either in the presence or absence of CHL. From passage 3, exponential cell cultures (OD_600_ of 1) were subjected to THP-1 infection assay. Alongside, overnight cultures of the *S. aureus* USA 300_LAC or JE2 (WT) and the autolysin transposon mutant strain, JE2 (*atl*::Tn) were also passaged similarly to the ppsf-CRISPRi strains without CHL and were sub-cultured into fresh TSB and grown to an OD_600_ of 1. Prepared *S. aureus* cells were diluted in PBS and adjusted to McFarland 0.5 and added to each well of seeded THP-1 macrophage cells at a multiplicity of infection (MOI) of 10. The host cells were infected for 1 hour, washed with PBS, and incubated in RPMI+ supplemented with 50μg/mL gentamicin to kill extracellular bacteria, and 10μg/mL CHL was added when appropriate.

For the time-course infection assay, at designated time points, the media were discarded, the mammalian cells were washed with PBS and lysed with 0.01% Triton X-100 in PBS, and the lysates were serially diluted and plated on TSA plates (supplemented with 10μg/mL CHL when appropriate). After 24 hours of incubation at 37°C, the bacterial colonies were counted.

For the time-course THP-1 cell cytotoxicity assay, at designated time points, the media were discarded, the cells were washed with PBS and MTT (3-(4,5-dimethylthiazol-2-yl)-2,5-diphenyltetrazolium bromide reagent, Invitrogen) was diluted (0.5mg/ml) in the RPMI+ cell culture media and added to the mammalian cells, followed by incubation at 37°C for 2 hours. The wells were washed twice with PBS, and dimethyl sulfoxide (DMSO) was added to solubilize the formazan crystals. Alongside these samples, control assays with uninfected cells (in the presence and absence of CHL) were conducted to ensure assay validity. The absorbance at 520 nm was measured using a BioTek Synergy H1 plate reader.

#### *Galleria mellonella* infection assay

For infection experiments, *S. aureus* ppsf-CRISPRi (NTC) strain, MR39 and ppsf-CRISPRi (*atl*) strain, MR40 (Table 2) were grown in TSB medium with CHL. The bacterial cells were then subjected to a three-step serial culture process in TSB medium with 24-hour intervals (denoted passages 1,2 and 3) in the absence of CHL. From passage 3, exponential cell cultures (OD_600_ of 1) were subjected to *Galleria* infection assay. Alongside, overnight cultures of the *S. aureus* USA 300_ LAC or JE2 (WT) and the autolysin transposon mutant strain, JE2 (*atl*::Tn) were also passaged similarly to the ppsf-CRISPRi strains without CHL and were sub-cultured into fresh TSB and grown to an OD_600_ of 1. Cells were washed twice in PBS, suspended in PBS, adjusted to McFarland 0.5, and diluted in PBS to a final concentration of 10^5^ cells/ml. Larvae of approximately equal weight were inoculated with 10 μL of this bacterial suspension, resulting in an infection dose of about 10^3^ cells/larva. Bacteria were injected into the hemocoel of the larvae between the last pair of legs using a 30-G syringe microapplicator (0.30 mm [30 G] × 8 mm, BD Micro-Fine Demi). As a control, larvae were mock-inoculated with 10 μL PBS. The larvae were then placed in 9.2 cm Petri dishes, and incubated at 37°C in darkness, and survival of the larvae was observed every 8 hours at 37°C. Larvae were regarded dead when they were not moving upon repeated physical stimulation. Each experiment was independently replicated at least three times using 3 biological replicates of each strain.

### Quantification and statistical analysis

Throughout all experiments conducted in this study, each condition was tested using a minimum of three biological replicates, each of which was tested as 2-3 corresponding technical replicates. For each biological replicate, mean values were calculated that were used for subsequent statistical analysis. Statistical significance was assessed using an unpaired, two-tailed Student’s *t*-test,two-way ANOVA, or log-rank (Mantel-Cox) test as appropriate. P values indicating significance are depicted as follows: ∗∗∗p < 0.001, ∗∗∗∗p < 0.0001, and ns is non-significant and is provided in the relevant figure legends. A *p*-value < 0.05 was considered statistically significant. Statistical analyses were performed with GraphPad Prism 10.0.0 for Windows (GraphPad Software, Boston, Massachusetts USA, www.graphpad.com). Results are presented as mean ± standard deviation (SD) in the figure legends.
