# Supplementary material for: A programmable, selection-free CRISPR interference system in Staphylococcus aureus for long-term host interaction studies
Source: iScience. 2025 Aug 21;28(9):113420. doi: 10.1016/j.isci.2025.113420 (PMC12447901; doi:10.1016/j.isci.2025.113420)
Supplement: Document S1. Figures S1–S5 and Tables S1–S3 [file mmc1.pdf]

iScience, Volume *28*

Supplemental information

A programmable, selection-free CRISPR interference system in *Staph-ylococcus aureus* for long-term host interaction studies

Roni Miah, Mona Johannessen, Morten Kjos, and Christian S. Lentz

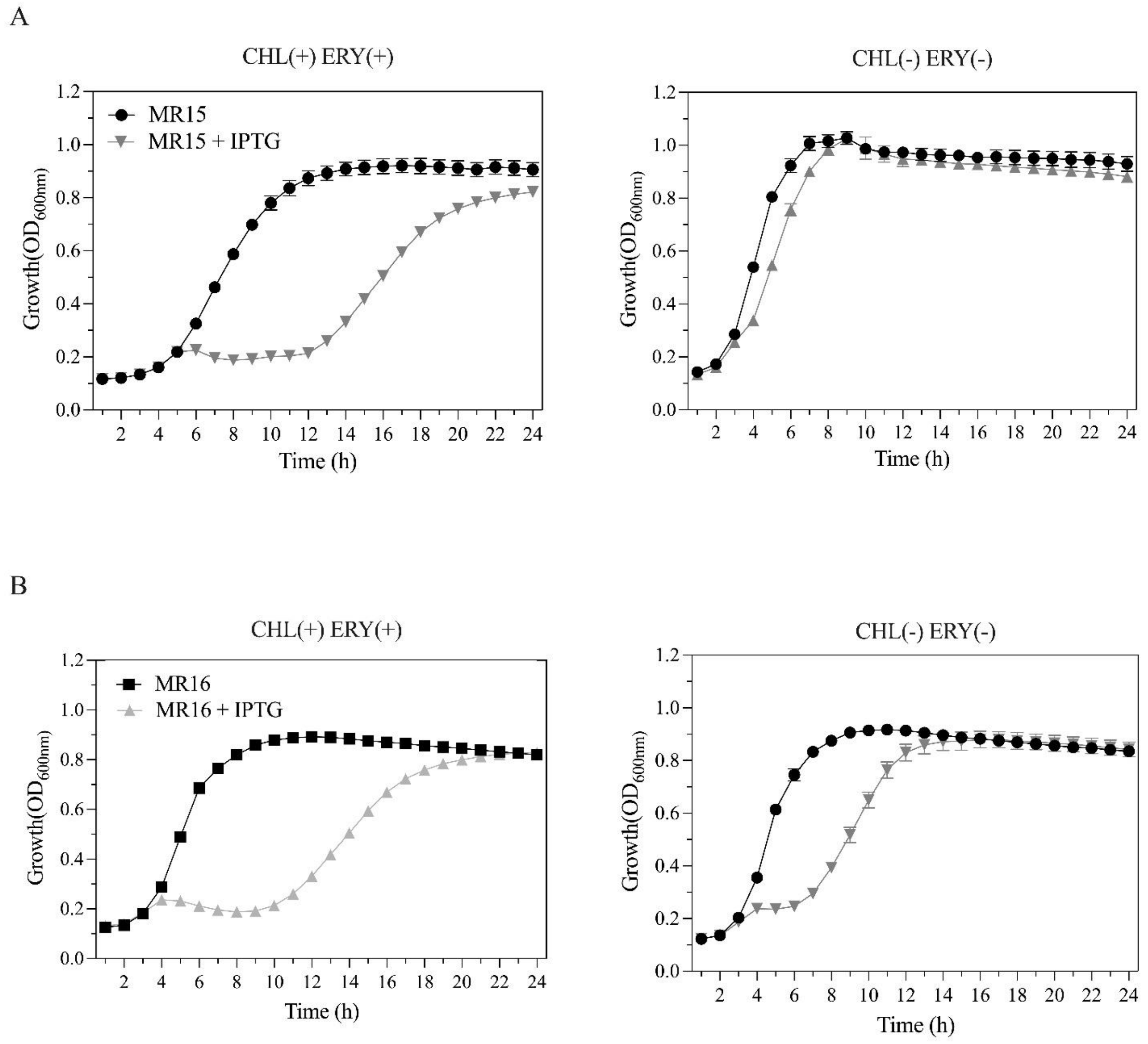

Figure S1. Antibiotic selection is essential for effectiveness of the classical CRISPRi system. Growth (OD_{600}) of IPTG-inducible CRISPRi strains targeting the essential peptidoglycan biosynthesis gene *pbp1*. The expression of dCas9 was regulated through IPTG induction via *lac* promoter on plasmid pLOW, while sgRNA is constitutively expressed from either pVL2336 (MR15, A) or pCM29 (MR16, B). Cells were grown in TSB with (+) and without (-) antibiotic selection in the presence or absence of IPTG (250 µM). Data show mean ± SD of n = 3 biological replicates (each recorded with three technical replicates). CHL; chloramphenicol, ERY; erythromycin.

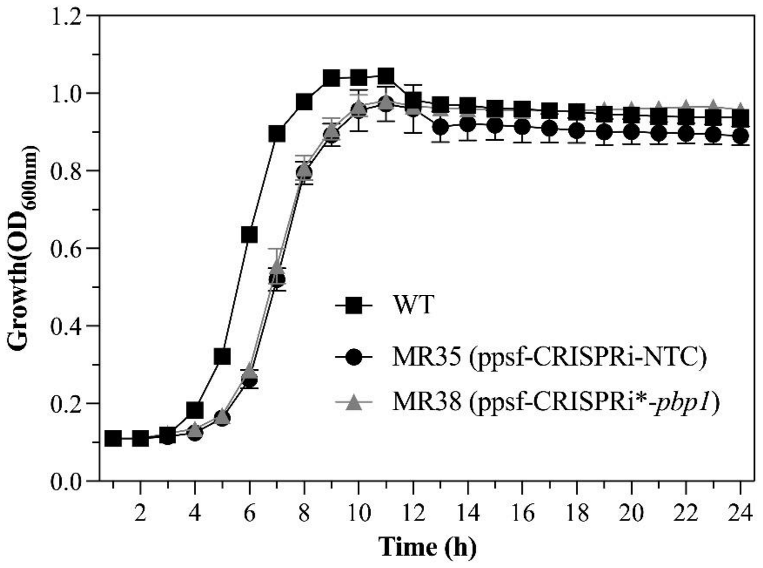

Figure S2. The ppsf-CRISPRi construct with a truncation in *dcas9* is not functional. Growth (OD_{600}) of the indicated *S. aureus*, WT, ppsf-CRISPRi strains grown in TSB without chloramphenicol for WT and under chloramphenicol selection for ppsf-CRISPRi strains. Full-length, 4107 bp (in MR35) or truncated *dcas9*, 559 bp (in MR38) expression was controlled by the P*coa* promoter. The sgRNAs targeted either the essential peptidoglycan biosynthesis gene *pbp1* to induce growth inhibition, or an NTC sequence derived from the luciferase gene. Data show mean ± SD of n = 3 biological replicates (each recorded with three technical replicates).

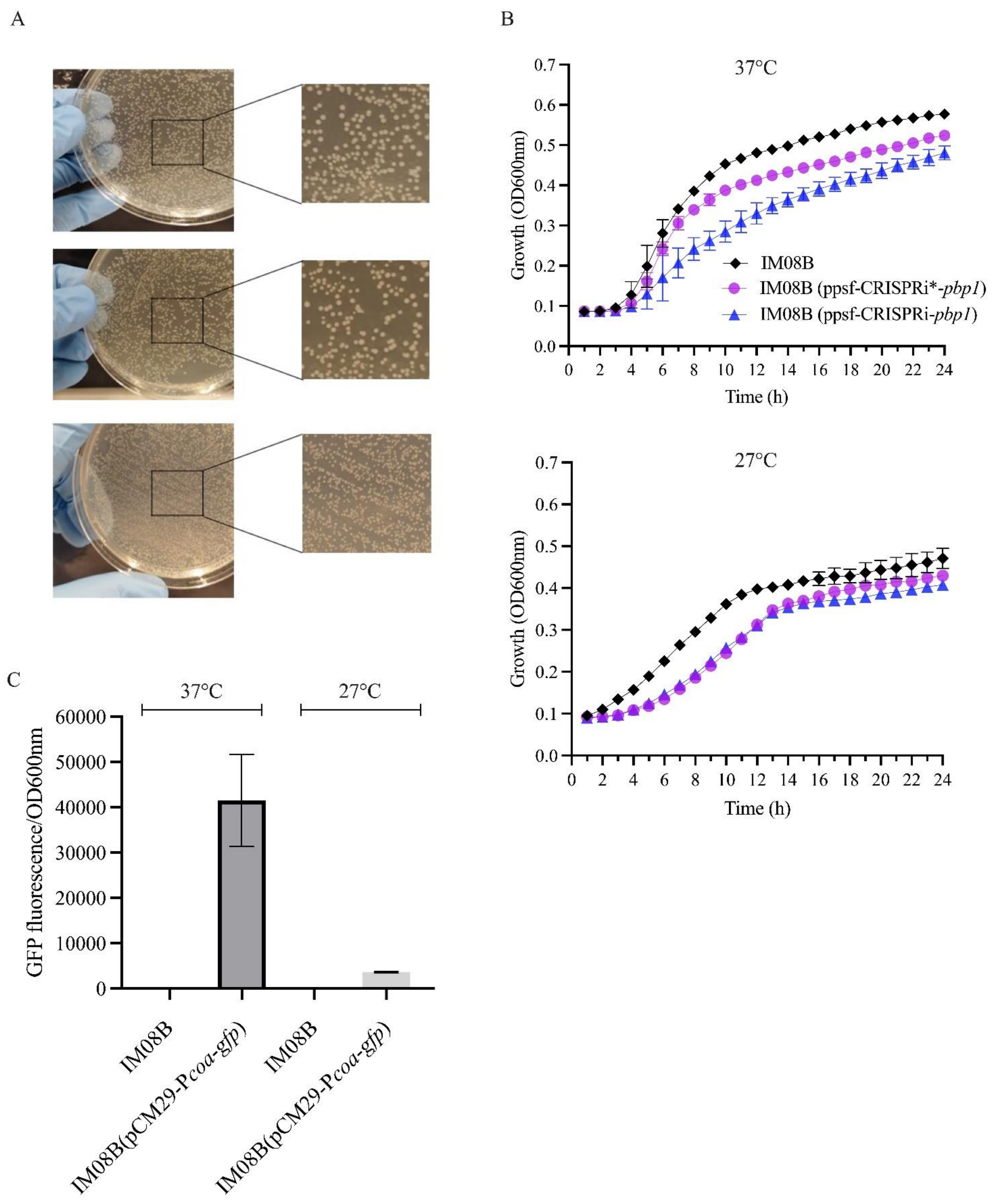

Figure S3. The toxicity of dCas9 is correlated with the level of dCas9 expression from ppsf-CRISPRi construct in *E. coli* IM08B. (A) Photographs of *E. coli* IM08B WT (top), IM08B transformants with ppsf-CRISPRi*(*pbp1*) (middle) and ppsf-CRISPRi (*pbp1*) (bottom) at 37°C. Small-colony phenotypes indicate cell toxicity (bottom). (B) Growth curves of *E. coli* IM08B (WT),

IM08B transformants with ppsf-CRISPRi*(*pbp1*) and ppsf-CRISPRi (*pbp1*) at 37°C and 27°C. ppsf-CRISPRi* construct's *dcas9* is truncated (559 bp) and nonfunctional. (C) *S. aureus* coagulase gene promoter (P*coa*) activity in *E. coli* IM08B at 37 and 27°C. *S. aureus* coagulase gene promoter-based fluorescent reporter plasmid (pCM29-P*coa-gfp*) was transformed into *E. coli* IM08B strains to check the level of functionality of that gene promoter by GFP fluorescence intensity measurement. GFP fluorescence data was normalized to the OD_{600} value of the corresponding sample. The *E. coli* IM08B strains without fluorescent reporter plasmid are included as controls. Bars show means ± standard deviation of n=3 biological replicates (each recorded with 3 technical replicates).

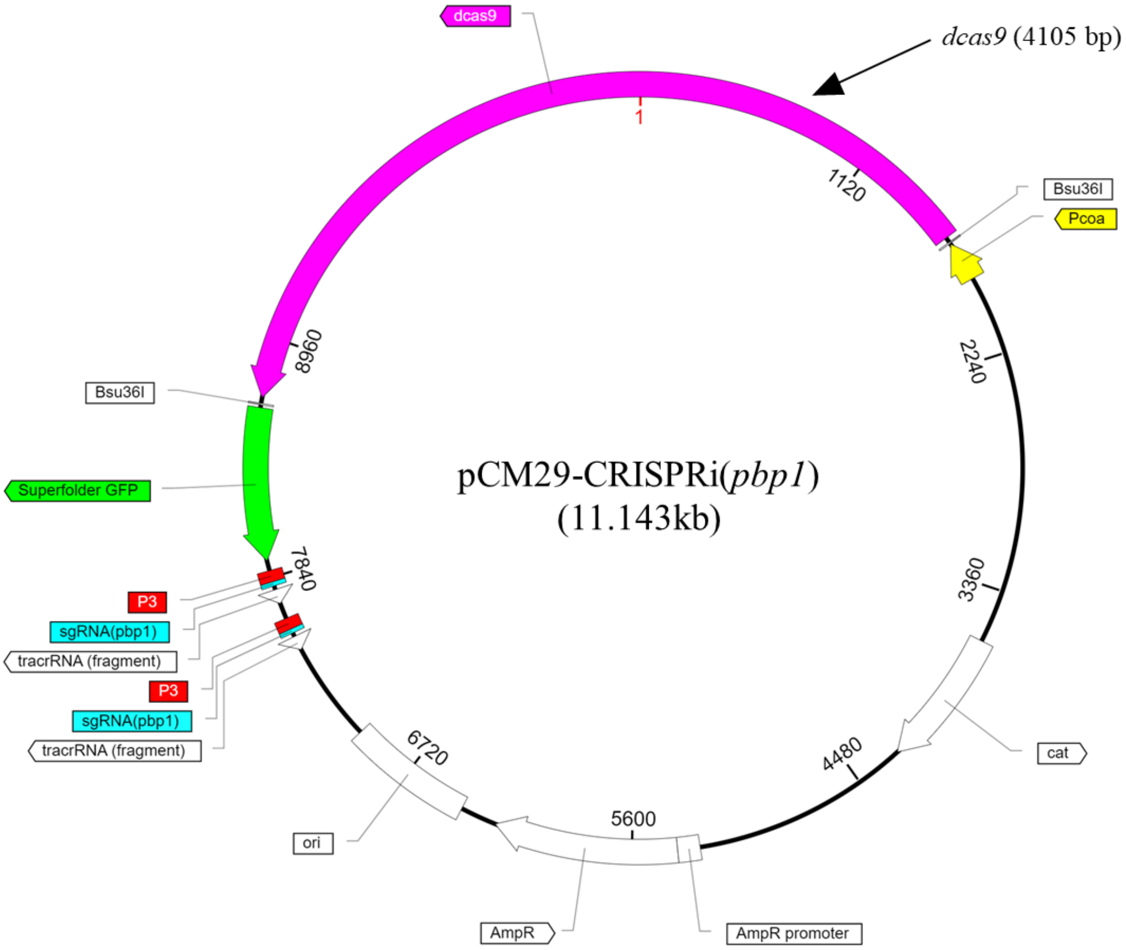

Figure S4. Stable ppsf-CRISPRi construct cloning in *E. coli* IM08B at 27°C. Plasmid map of ppsf-CRISPRi(*pbp1*), cloned in *E. coli* IM08B at 27°C. The plasmid was sequenced using nanopore techniques developed by Eurofins (https://eurofinsgenomics.eu/en/custom-dnasequencing/eurofins-services/whole-plasmid-sequencing/), deposited in Addgene (https://www.addgene.org/241716/), and the plasmid map was created using SnapGene based on the provided whole plasmid sequence.

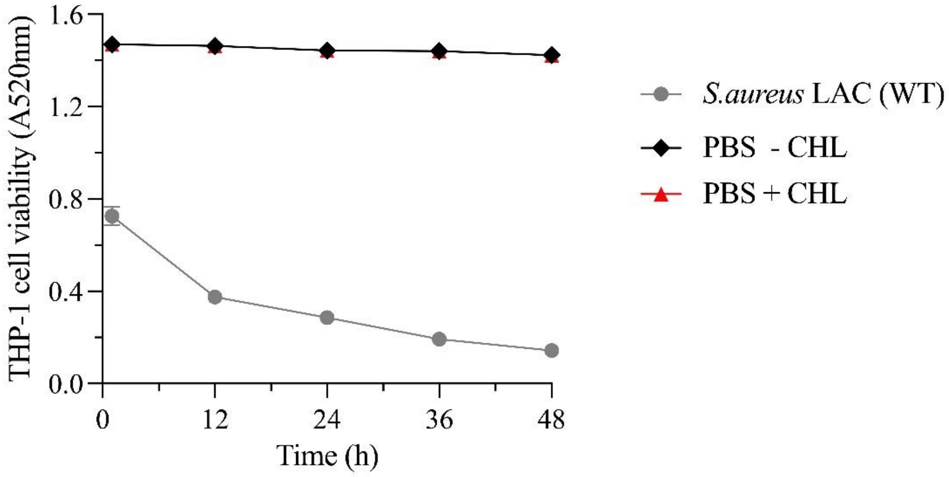

Figure S5. Chloramphenicol does not affect the viability of THP1 cells. Time-course THP-1 cell viability assay following infection with WT *S. aureus* USA 300 LAC strain or with PBS with (+) or without (-) CHL. THP-1 cells were incubated in RPMI+ with 50 µg/ml Gentamicin. The data represents the mean ± standard deviation of three biological replicates.

Table S1 Plasmids used in this study

Plasmids	Relevant characteristics[a]	Source or reference
pCM29-P*coa-gfp*	pCM29 carrying *gfp* downstream of *S. aureus* coagulase gene promoter[b], amp^R, cm^R	[50]
pLOW- P*lac-dcas*9	pLOW carrying mutated Cas9 (*dcas9)* downstream of *lac* promoter, amp^R, ery^R	[18]
pVL2336-sgRNA (*pbp*1)	pVL2336 carrying sgRNA*(pbp1*) expression cassette, amp^R, cm^R	[18]
pCM29-sgRNA (*pbp*1)	pCM29 carrying sgRNA(*pbp1*) expression cassette, amp^R, cm^R	[50]
ppsf-CRISPRi constructs	pCM29 carrying *dcas9*, *gfp* downstream of *S. aureus* coagulase or autolysin gene promoter[b], and sgRNA expression cassette under constitutive P3 promoter, amp^R, cm^R	This study
P*coa*-TruncCRISPRi (*pbp1*)	sgRNA target *pbp1*	This study https://www.addgene.org/241712/
P*coa*-CRISPRi (NTC)	sgRNA target NTC	This study https://www.addgene.org/241713/
P*coa*-CRISPRi (*coa*)	sgRNA target *coa*	This study https://www.addgene.org/241714/
P*coa*-CRISPRi (*atl*)	sgRNA target *atl*	This study https://www.addgene.org/241715/
P*coa*-CRISPRi (*pbp1*)	sgRNA target *pbp1*	This study https://www.addgene.org/241716/
P*atl*-CRISPRi (NTC)	sgRNA target NTC	This study https://www.addgene.org/241717/
P*atl*-CRISPRi (*atl*)	sgRNA target *atl*	This study https://www.addgene.org/241718/

[a] amp^R, ampicillin resistance; cm^R, chloramphenicol resistance; ery^R, erythromycin resistance; NTC, non-target control; Trunc, truncated *dcas9*

[b] Promoter sequences were selected as the non-coding gap sequence between the gene and its upstream gene.

Table S2 Primers used in this study

Name	Sequence 5`-> 3`
RM 27_ *dcas9*_Bsu36I F	CGC**CCTTAGG**GGTACCAAAGAG
RM 28_ *dcas9*_Bsu36I R	CGC**CCTTAGG**GGCCAGTGAATT
RM 29_q*gfp*_F	GCACTACTGGAAAACTACCTGT
RM 30_q*gfp*_R	CTGTACATAACCTTCAGGCATGGCA
RM 21_q*coa* F	GACCGCAATTTAACAAAACACC
RM 22_ q*coa* R	AGCTCCGTATGATACTTGACC
RM 31_ q*groE*_F	TGGCTAACACGTGCATCAAT
RM 32_ q*groE*_R	AAAAGCACCTGGTTTTGGTG
RM 19_ q*rrsA* F	ACGGTCTTGCTGTCACTTATA
RM 20_ q*rrsA* R	TACACATATGTTCTTCCCTAATAA
MK25_sgRNA check R	AAATCTCGAAAATAATAGAGGGA
MK 26_sgRNA check F	GGATAACCGTATTACCGCCT
MK 44_*dcas9* check R	TGTCCGTTTGAGACGAGTC
RM 17_*gfp* check R	GTTACAAACTCAAGAAGGACC

gfp, green fluorescent protein; F, Forward; R, Reverse

Table S3 Sequences of sgRNA base pairing regions

Target	Sequence 5`-> 3`
pbp1	GAACGAGGAAAGATATATGA
coa	TGGAGATACAGACAATCCAC
atl	AGCGTTAATGCAACCATTGA
no-target control, NTC	CGGCGCCATTCTATCCTCTA
